# Repositioning of moxidectin: a promising approach in cutaneous leishmaniasis therapy

**DOI:** 10.1051/parasite/2025035

**Published:** 2025-07-04

**Authors:** Lynn Al Samra, Mohamad El Nahas, Ilham Mneimneh, Sima Tokajian, Georges Nemer, Aia Sinno, Kelven Rahy, Sergio Thoumi, Zahraa Zibara, Ahmad El Khatib, Dalal Sabbagh, Jacques Guillot, Louna Karam, Lazo Ali, Ruqaya Baghdadi, Charbel Al Khoury

**Affiliations:** 1 Department of Natural Sciences, School of Arts and Sciences, Lebanese American University Beirut Campus P.O. Box 13-5053 Chouran 1102 2801 Beirut Lebanon; 2 Department of Computer Science and Mathematics, Lebanese American University Beirut Campus P.O. Box 13-5053 Chouran 1102 2801 Beirut Lebanon; 3 Department of Natural Sciences, School of Arts and Sciences, Lebanese American University Byblos Campus P.O. Box 36 Byblos Lebanon; 4 Division of Genomics and Translational Biomedicine, College of Health and Life Sciences, Hamad Bin Khalifa University P.O. Box 34110 Doha Qatar; 5 Gilbert and Rose-Marie Chagoury School of Medicine, Lebanese American University Byblos Campus P.O. Box 36 Byblos Lebanon; 6 Oniris VetAgroBio, École Nationale Vétérinaire, Agroalimentaire et de l’Alimentation Nantes-Atlantique Campus de la Chantrerie, 101 Route de Gachet 44307 Nantes Cedex 3 France; 7 IRF, IRCAT, Université d’Angers, UBO, Institut de Biologie en Santé – PBH-IRIS, CHU 4 Rue Larrey 49933 Angers Cedex 9 France

**Keywords:** *Leishmania*, leishmaniasis, Moxidectin, Drug repurposing, RNA-Seq, Drug resistance

## Abstract

Cutaneous leishmaniasis presents a significant challenge to public health due to its diverse clinical manifestations, resistance development, and treatment-related adverse effects. Here, we examined the efficacy of ivermectin, moxidectin (MOX), afoxolaner, and permethrin against all stages of *Leishmania tropica* and THP-1 cells. We also assessed the potential for resistance acquisition after 15 rounds of artificial selection. To elucidate the mode of action of MOX, we employed RNA sequencing, molecular dynamics simulation, and chloride flux assays. Additionally, we evaluated the therapeutic index of MOX using the *Galleria mellonella* infection model. MOX demonstrated the highest selectivity index against leishmaniasis (promastigotes: 0.58 μM; amastigotes: 0.96 μM; host cells: 60.29 μM). Moreover, MOX exhibited the lowest resistance acquisition in both promastigotes and intracellular amastigotes after 15 rounds of artificial selection, with resistance ratios of 17.23 and 4.59, respectively. Post-exposure to MOX, differential gene expression profiles showed both stage-specific and stage-unspecific enrichment of gene families involved in crucial biological processes. Moreover, molecular dynamics simulations revealed a potential neutralizing effect of MOX on the chloride channel of *L. tropica*. Specifically, MOX binds to the selectivity filter, potentially disrupting the osmotic equilibrium and thereby killing the parasite. The *in vivo* introduction of MOX significantly inhibited the growth of *L. tropica* in *G. mellonella* larvae, resulting in decreased rates of mortality and melanization. These findings indicate that MOX is a promising candidate for the treatment of *L. tropica* infections, warranting further investigation and potential consideration for clinical use*.*

## Abstract (fr)

La leishmaniose cutanée représente un défi majeur de santé publique en raison de la diversité de ses manifestations cliniques, du développement de résistances et des effets indésirables liés au traitement. Nous avons examiné l’efficacité de l’ivermectine, de la moxidectine (MOX), de l’afoxolaner et de la perméthrine contre tous les stades de *Leishmania tropica* et les cellules THP-1. Nous avons également évalué le potentiel d’acquisition de résistance après 15 cycles de sélection artificielle. Pour élucider le mode d’action de la MOX, nous avons utilisé le séquençage de l’ARN, la simulation de dynamique moléculaire et des dosages de flux de chlorure. De plus, nous avons évalué l’indice thérapeutique de la MOX à l’aide du modèle d’infection chez *Galleria mellonella*. La MOX a montré l’indice de sélectivité le plus élevé contre la leishmaniose (promastigotes : 0,58 μM; amastigotes : 0,96 μM; cellules hôtes : 60,29 μM). De plus, la MOX a montré la plus faible acquisition de résistance chez les promastigotes et les amastigotes intracellulaires après 15 cycles de sélection artificielle, avec des ratios de résistance respectivement de 17,23 et 4,59. Après exposition à la MOX, les profils d’expression génique différentiels ont montré un enrichissement spécifique et non spécifique au stade des familles de gènes impliquées dans des processus biologiques cruciaux. De plus, les simulations de dynamique moléculaire ont révélé un effet neutralisant potentiel de la MOX sur le canal chlorure de *L. tropica*. Plus précisément, la MOX se lie au filtre de sélectivité, perturbant potentiellement l’équilibre osmotique et tuant ainsi le parasite. L’introduction *in vivo* de MOX a significativement inhibé la croissance de *L. tropica* chez les larves de *G. mellonella*, entraînant une diminution des taux de mortalité et de mélanisation. Ces résultats indiquent que la moxidectine est un candidat prometteur pour le traitement des infections à *L. tropica*, justifiant des recherches plus approfondies et une éventuelle utilisation clinique.

## Introduction

Leishmaniases comprise a group of diseases caused by *Leishmania* spp. protozoan parasites, affecting various mammals, including humans [68]. According to the World Health Organization, over one million cases occur annually, with more than a billion people at risk in endemic regions [75]. Although leishmaniasis affects diverse socioeconomic groups, it is more prevalent in poverty-stricken areas due to factors like inadequate housing, migration, crowded living conditions, poor sanitation, and lack of personal protection measures [75]. The disease is primarily transmitted by phlebotomine sandflies, with over 90 species capable of spreading *Leishmania* through bites [25]. This broad distribution and the potential for disease outbreaks make leishmaniasis a significant public health concern, especially in regions with limited access to healthcare. Among the various forms of leishmaniasis, cutaneous leishmaniasis (CL) is the most prevalent, accounting for the majority of reported cases. It is characterized by ulcerative skin lesions that develop at the site of infection and can evolve into nodular plaques within 4–12 weeks [30]. These lesions, although rarely life-threatening, cause significant morbidity, scarring, and disfigurement, often leading to social stigma and reduced quality of life for affected individuals. In the Middle East, Asia, and Europe, *Leishmania tropica* is the primary cause of CL, and recent geopolitical changes, such as the influx of refugees into non-endemic areas, have contributed to a surge in cases, underscoring the urgent need for effective treatment options [6, 63]. Unlike *L. major*, which often leads to self-healing lesions, *L. tropica* infections are notoriously chronic, difficult to treat, and frequently lead to non-healing or relapsing lesions [11]. Current treatments for CL include pentavalent antimonials, amphotericin B, paromomycin, pentamidine, and miltefosine, which have been the mainstay of therapy for years [33, 70]. However, these treatments often present significant challenges, such as high toxicity, lengthy treatment courses, painful administration, and high costs, limiting their accessibility and adherence among patients, particularly in resource-poor settings [43]. Furthermore, increasing reports of drug resistance further compromise their efficacy, with *Leishmania* spp. demonstrating an alarming ability to adapt and develop resistance to multiple therapeutic agents, which greatly diminishes the available treatment options [44]. Given these limitations, there is a pressing need for safer, more effective, and accessible therapies to manage CL, particularly for populations in endemic areas who are most affected [75]. Drug repurposing has emerged as a promising strategy to address this gap by leveraging existing medications with established safety profiles to expedite the development of new therapies. This approach offers significant advantages, such as reduced development timelines, lower costs, and an already-known safety profile, making it especially valuable for neglected tropical diseases like leishmaniasis [58]. While repurposing has been successful with drugs like pentamidine and amphotericin B in treating leishmaniasis, the systematic exploration of repurposing antiparasitic agents for CL has been limited, particularly for *L. tropica* [13, 60]. Addressing this gap is critical, as it could lead to the discovery of effective treatments that are both more affordable and accessible for those in need.

To fill this gap, this study aimed to evaluate the efficacy of commonly used antiparasitic drugs – permethrin (PER), ivermectin (IVM), moxidectin (MOX), and afoxolaner (AFO) – against *L. tropica*. These drugs were selected based on their established effectiveness against various parasitic infections and their potential modes of action against *Leishmania* spp. [22, 48]. IVM and MOX, for example, are macrocyclic lactones known for their strong antiparasitic activity, while AFO, an isoxazoline, has demonstrated efficacy against a broad range of ectoparasites and protozoans. As *Leishmania* spp. are known for their adaptability, it is crucial to investigate how *L. tropica* responds to these repurposed drugs to assess their potential for long-term use and to prevent future resistance.

To comprehensively understand their mode of action and resistance potential, we used RNA sequencing (RNA-Seq), which provides a detailed analysis of gene expression changes in response to drug exposure, offering insights into the molecular mechanisms driving the drugs’ effects [50]. This technique allows us to identify specific pathways and genes targeted by the drugs, contributing to a more precise understanding of their antiparasitic action. Additionally, the *Galleria mellonella* wax moth larva served as an effective *in vivo* model due to similarities to mammalian immune responses, ease of handling, and cost-effectiveness. Its innate immune system, which includes cellular and humoral defense mechanisms, makes it a valuable model for studying infection dynamics and drug efficacy [45]. Using *G. mellonella* allows us to evaluate the therapeutic index of these drugs in a more complex biological context, providing a valuable bridge between *in vitro* studies and more traditional mammalian models. By combining these methodologies, this study aimed to evaluate the efficacy, mode of action, and potential resistance acquisition of these repurposed drugs against *L. tropica*, ultimately identifying promising candidates for advancing CL therapy. This comprehensive approach will not only address the current treatment gaps, but will also contribute to the development of more effective and sustainable treatment strategies for CL.

## Materials and methods

### Ethics

Ethical approval was obtained from the Institutional Review Board (IRB) at the American University of Beirut Medical Center (AUBMC) on August 1, 2011, prior to sample collection (Approval Ref. No. PALM I.K.01).

### Molecule to be evaluated

PER (Sigma-Aldrich, St. Louis, MO, USA), IVM (Sigma-Aldrich), MOX (Sigma-Aldrich), AFO (LGC Standards, Teddington, UK), and miltefosine (ML) (Sigma-Aldrich) were stored following the guidelines specified by the respective manufacturers until their subsequent utilization.

### Parasite culture and maintenance

*Leishmania tropica* LT2 (strain designation: MHOM/LB/2015/IK) was isolated in 2014 from skin punch biopsies collected at the American University of Beirut Medical Centre (AUBMC), as described previously [63]. Promastigotes were cultured in RPMI-1640 medium supplemented with 20% heat-inactivated fetal bovine serum (FBS) and 1% penicillin-streptomycin, and incubated at 25 °C in a 5% CO_2_ environment. Promastigotes were then used to generate axenic amastigotes following the method outlined by Teixeira *et al.* [71]. THP-1 cells were maintained in RPMI-1640 medium supplemented with 20% FBS and 1% penicillin-streptomycin at 37 °C in a 5% CO_2_ incubator. To differentiate THP-1 cells into an adherent macrophage-like phenotype, 1 million cells per well were seeded into 6-well plates and treated with 25 ng/mL phorbol 12-myristate 7-acetate (PMA). Following PMA treatment, adherent cells were washed three times with phosphate-buffered saline (PBS) and replenished with a fresh culture medium. Macrophage activation was achieved by adding lipopolysaccharide (LPS) derived from *Escherichia coli* 0111 at a final concentration of 1 ng/mL for 4 h. For the cultivation of intracellular amastigotes, THP-1 cells were infected with metacyclic promastigotes at a multiplicity of infection (MOI) ratio of 1:10 (host:parasite) and incubated for 48 hours at 37 °C in a 5% CO_2_ incubator. Non-internalized promastigotes were removed by washing the infected cells three times with PBS.

### Efficacy of antiparasitic drugs against different developmental stages of *L. tropica* and THP-1 cells

One million THP-1 cells, as well as equivalent numbers of *L. tropica* promastigotes, axenic amastigotes, and intracellular amastigotes, were cultured in microplates (1 × 10^6^ cells/well in 2 mL RPMI) and treated with a range of repurposed drug concentrations (0.01, 0.05, 0.1, 0.5, 1, 2, 5, 7, and 10 μM) for 72 h. ML, a molecule with known anti-leishmanial activity, served as a positive control. Cell replication was assessed using Real Time-quantitative Polymerase Chain Reaction (RT-qPCR) for absolute quantification of gene expression, following the method described previously [2]. Briefly, total RNA was extracted from both treated and untreated control cells using an RNeasy Mini Kit (QIAGEN, Hilden, Germany), according to the manufacturer’s instructions, followed by DNase I treatment (Fermentas, #EN0521, 37 °C for 30 min) to eliminate genomic DNA contamination. RNA purity was assessed using a NanoDrop spectrophotometer (260/280 nm ratio), and integrity was verified via 1.5% agarose gel electrophoresis. Subsequently, 2 μg of extracted RNA was reverse transcribed into complementary DNA (cDNA) using a RevertAid First cDNA Synthesis Kit (Thermo Scientific, Waltham, MA, USA). Gene expression was assessed using SYBR^®^ Green 2× (Sigma Aldrich) in a CFX96 Real-Time PCR System (Bio-Rad, Hercules, CA, USA). *Leishmania* replication was quantified by amplifying the kDNA minicircle, while GAPDH served as the reference gene for THP-1 cells. The kDNA primers used for *L. tropica* amplification were forward (600 nM): 5′-CCTATTTTACACCAACCCCCAGT-3′ and reverse (300 nM): 5′-GGGTAGGGGCGTTCTGCGAAA-3′, generating a 116 bp amplicon. For macrophages, GAPDH primers were used at 300 nM each, with forward: 5′-TCCCACCTTTCTCATCCAAG-3′ and reverse: 5′-CATCACCCCTCTACCTCCCT-3′, generating a 128 bp amplicon. Each 25 μL reaction contained 12.5 μL of SYBR Green Master Mix, 1 ng of cDNA, optimized forward and reverse primers, and nuclease-free water. The cycling conditions were: 95 °C for 1.5 min, followed by 40 cycles of 95 °C for 15 s, 59.2 °C (kDNA) or 58.2 °C (GAPDH) for 30 s, and 72 °C for 30 s, with melting curve analysis confirming specificity. Absolute quantification was performed using a standard curve approach, where dsDNA standards were prepared from purified PCR products and serially diluted. The Ct values of experimental samples were plotted against these standard curves to determine gene expression levels. Negative controls (no-template controls, NTCs) were included to check for contamination [2]. The half-maximal inhibitory concentration (IC_50_) was calculated for each drug, with experiments performed in triplicate and repeated three times.

### Resistance selection assay

The *L. tropica* strain initially identified as susceptible (LS-LT2) was cultured without exposure to any antiparasitic drugs. To induce drug resistance, LS-LT2 was gradually exposed to increasing concentrations of each drug, ranging from 0 to 50 μM. At each generation, the drug concentration was adjusted to maintain a selective pressure that resulted in approximately a 50% reduction in the parasite population [4]. Every five generations, the efficacy of the drugs against different developmental stages of *L. tropica* was evaluated, and IC_50_ was determined. After 15 generations under this selection pressure, strains resistant to specific drugs were established and designated as follows: PER-resistant LT2 (PERR-LT2), IVM-resistant LT2 (IVMR-LT2), MOX-resistant LT2 (MOX-RLT2), AFO-resistant LT2 (AFO-RLT2), and ML-resistant LT2 (ML-RLT2). All experiments were conducted in triplicate and repeated three times to ensure the robustness and reproducibility of the results.

### *In vitro* resistance study: prolonged exposure of promastigotes

Promastigotes were cultured at a concentration of 1 × 10^6^ cells/mL in 10 mL of RPMI-1640 medium supplemented with 5% FBS. To apply selection pressure, incremental drug doses were administered to achieve a 50% reduction in the promastigote population. After 72 hours of initial exposure, promastigotes were transferred into a fresh medium and incubated for an additional 72 hours. The cell concentration was then adjusted back to 1 × 10^6^ cells/mL, and the culture was transferred to a new flask for continued drug exposure in the next cycle.

### *In vitro* resistance study: prolonged exposure of intracellular amastigotes

Intracellular amastigotes were seeded in 6-well plates at a concentration of 1 × 10^6^ cells/mL and exposed to drug concentrations that induced a 50% reduction in the parasite population. Following drug exposure, the plates were washed three times with PBS to remove residual drugs. To recover and transform the surviving parasites, 20 μL of RPMI-1640 medium containing 0.05% sodium dodecyl sulfate (SDS) was added, as described previously [32]. After a 30-second incubation, 180 μL of RPMI-1640 medium supplemented with 10% FBS was added to each well, allowing controlled lysis of the infected THP-1 cells while preserving the viability of the parasites [32]. The plates were then incubated at 27 °C for 48 h to facilitate the transformation of intracellular amastigotes into promastigotes. Once promastigote growth was identified, the parasites were further amplified in a 25 mL culture flask. The resulting metacyclic promastigotes were used for subsequent infection cycles in macrophages, where intracellular amastigotes were re-exposed to the drug regimen.

### RNA-Seq and transcriptome analysis

To elucidate the mode of action of MOX, the drug with the highest selectivity index, RNA-Seq analysis was conducted on MOX-treated and untreated promastigotes and intracellular amastigotes, using three biological replicates. Total RNA was extracted with an RNeasy Mini Kit (QIAGEN), following the manufacturer’s protocol, and residual genomic DNA was removed with DNase I treatment. RNA quality and concentration were verified using a Nanodrop 1000 and Bioanalyzer (Agilent 2100, Agilent, Santa Clara, CA, USA). RNA samples were then sent to Macrogen (Seoul, Republic of Korea) for paired-end sequencing on the HiSeq 4000 platform, with poly-A enrichment to generate strand-specific cDNA libraries. Low-quality reads and adapter sequences were removed using Trimmomatic (v0.39) [15], and read quality was checked with FastQC (v0.11.9) [8]. Reads with an average quality score below 15 or length under 36 bp were excluded. For RNA-Seq analysis, reads were aligned to the *L. tropica* LT2 reference genome using TopHat2 v2.1.1 [36]. Transcriptome reconstruction was done with Cufflinks v2.2.1 [72], and differential gene expression was analyzed using Cuffdiff v2.2.1 [72]. Differentially expressed genes (DEGs) were identified as those with an FPKM value > 1, a log2 fold change > 0, and a *p*-value < 0.05. Gene set enrichment analysis (GSEA) was conducted using RStudio (version 4.3.0) to identify dysregulated biological processes and pathways following MOX exposure in *L. tropica* promastigotes and intracellular amastigotes. Gene sets were created by integrating differentially expressed genes (DEGs) from transcriptomic profiling with Gene Ontology (GO) annotations (Supplementary Note 1). Overrepresented GO terms among the DEGs were identified, and genes with identical GO annotations were grouped into functionally coherent gene sets [69]. This systematic approach organized DEGs based on biological processes, molecular functions, and cellular components according to the GO knowledgebase [9].

### Chloride flux assay

Chloride ion concentration in promastigotes was measured using a chloride assay kit (ab83372, Abcam, Cambridge, UK), according to the manufacturer’s guidelines, in the presence and absence of MOX. Promastigotes were treated with the IC_50_ concentration of MOX for 24 hours. Subsequently, one million cells were homogenized in 100 μL of lysis buffer (pH 6.5–8.0) and centrifuged at 13,000 rpm for 10 min to remove insoluble material. The supernatant was diluted with assay buffer, and 50 μL of the sample was adjusted to the well volume with distilled water. Then, 150 μL of chloride reagent was added to each well, and after a 15-minute incubation at room temperature, optical density (OD) at 620 nm was measured using a Varioskan^TM^ LUX multimode microplate reader (Thermo Scientific). Negative (culture medium without drugs) and positive controls (anthracene-9-carboxylic acid), a known chloride channel inhibitor in *Leishmania* spp. were included in the assay [56]. The experiment was performed in triplicate and repeated three times to ensure accuracy.

### Building the simulation system

The ligand MOX was obtained from PubChem in .sdf format and converted into a three-dimensional (3D) structure for docking. A homology-based model of the CLC protein was generated using SWISSMODEL, with the CLC protein (E9AHM3.1.A) serving as a template. The model was refined based on the MolProbity score. Molecular docking was conducted using AutoDock-GPU v4.2.6, examining the selectivity filter, ligand binding site I, and ligand binding site II as potential MOX binding sites, following established protocols [21]. The known CLC inhibitor 9-anthracene-carboxylic acid (9-AC) [74] and activator Lubiprostone [49] served as positive controls. The conformation with the lowest binding energy was selected for further dynamic simulations.

A 100 ns molecular dynamics (MD) simulation was conducted using GROMACS v.2022.4 with GPU acceleration to investigate ligand-macromolecule interactions [1, 5]. Protein topologies were generated using the CHARMM36 force field, and ligand topologies were converted via CGENFF scripts from the Mackerell laboratory. Complexes were placed in a dodecahedral unit cell with the SPC model for water, and ions were added for neutralization. Energy minimization, NVT (2 ns), and NPT (8 ns) ensembles were executed, followed by the 100 ns simulation using the leapfrog method. Root mean square fluctuation (RMSF) and root mean square deviation (RMSD) were analyzed, and binding free energy was calculated using the MM/PBSA method [37] with the g_mmpbsa tool in GROMACS [38].

### *Galleria mellonella* infection model

In this investigation, *G. mellonella* was used as an insect model to assess the therapeutic efficacy of drugs against leishmaniasis. Larvae were collected from a beehive and maintained on a diet of wax and honey under controlled conditions at 28 °C in an oxygenated incubator. To ensure uniformity, larvae with consistent pale pigmentation and an average weight of approximately 300 mg were selected for experiments. Hemolymph was collected by puncturing a larval proleg with a Hamilton syringe, followed by gentle pressure to extract the hemolymph, which was chilled on ice and mixed with 500 μL of insect physiological saline (IPS). The collected hemolymph was centrifuged, washed twice with 500 μL of ice-cold IPS, and resuspended in 1,000 μL of IPS. Hemocyte counts were determined using a Neubauer chamber. For infection, larvae were placed in a dorsal position, their abdomens sterilized with 70% ethanol and then inoculated with *L. tropica* metacyclic promastigotes at a ratio of 1:10 (host cell: parasite). Based on allometric scaling, MOX (0.2 mg/kg) or ML (2.5 mg/kg) was administered to the infected larvae. Parasite proliferation was monitored over 14 days, with RT-qPCR analysis conducted to quantify the parasite load. Hemocoel contents (including hemocytes and *Leishmania*) were extracted from three infected larvae per group after incubation at 37 °C, and the RT-qPCR assay was performed in duplicate, with the entire experiment repeated 10 times. Larval behavior, including mortality and melanization, was monitored over the 14-day period. Larvae were considered dead if immobile and unresponsive to stimuli, while melanization was indicated by dark surface coloration. The experiment included four groups: RPMI-infected larvae (placebo control), promastigote-infected larvae (PIL), PIL treated with ML, and PIL treated with MOX, with each group undergoing 10 independent experiments using 10 larvae per trial.

### Statistical analysis

IC_50_ values and their standard errors were calculated using probit regression in SPSS Statistics for Windows, version 25.0 (IBM Corp., Armonk, NY, USA). To compare the efficacy of the drugs against all developmental stages of the parasite and THP1 cells, a one-way ANOVA was performed, followed by a post-hoc Tukey’s Honest Significant Difference (HSD) test to identify statistically significant differences between groups. The same statistical approach was used to assess the selectivity index of the drugs against intracellular amastigotes, the clinically relevant stage of *L. tropica*. To further evaluate differences in selectivity, Cohen’s d effect size analysis was conducted to measure the magnitude of the differences between drugs. Additionally, Bayesian analysis was applied to quantify the strength of evidence supporting the observed differences. To assess differences in resistance development across the repositioned drugs, resistance ratios (RRs) were calculated by dividing the IC_50_ value of each resistant strain, after 15 rounds of selection pressure, by the IC_50_ value of the original LS-LT2 strain to quantify the level of resistance acquired. To statistically compare the resistance acquisition across different drugs, one-way ANOVA was performed separately for promastigotes and amastigotes, using the RR values. Differentially expressed gene sets were identified using GSEA in RStudio version 4.3.0, with significant gene expression changes defined as adjusted *p* < 0.05 and log_2_ fold change (FC) < 0 for downregulated or > 0 for upregulated genes. To evaluate MOX’s ability to modulate CLCs, one-way ANOVA followed by a post-hoc Tukey’s HSD test was performed to compare means, with a significance level set at 5%. The *in vivo* efficacy of MOX in *G. mellonella* was assessed by calculating the area under the curve (AUC) for each larva using the trapezoidal rule, integrating the log concentration of *L. tropica* over the 14-day period. Differences in AUC between groups were analyzed using one-way ANOVA, followed by post-hoc Tukey’s HSD test. Larval survival and melanization were analyzed using Kaplan-Meier survival curves, and statistical comparisons between groups were performed using the log-rank (Mantel-Cox) test, a non-parametric method appropriate for time-to-event data. The resulting test statistic is reported as a Chi-square value, which reflects differences in the survival or melanization dynamics between groups. This method is appropriate for categorical time-based outcomes such as larval death or visible melanization. A *p*-value of ≤ 0.05 was considered statistically significant.

## Results

### Efficacy of antiparasitic drugs against all developmental stages of *L. tropica* as well as THP-1 cells

The efficacy of PER, IVM, MOX, AFO, and ML against *L. tropica* was assessed across promastigotes (*p* < 0.05; *F* value = 458.074; df = 4), axenic amastigotes (*p* < 0.05; *F* value = 195.488; df = 4), intracellular amastigotes (*p* < 0.05; *F* value = 119.226; df = 4), as well as their cytotoxicity on THP-1 cells (*p* < 0.05; *F* value = 104.747; df = 4) [2]. All drugs exhibited a dose-dependent effect on every developmental stage of the parasite (Table 1). In promastigotes, MOX, AFO, and IVM exhibited the greatest potency, showing significantly lower IC_50_ values compared to PER and ML, which were the least effective. Similarly, in axenic amastigotes, MOX demonstrated the strongest activity, followed by IVM and AFO, while PER and ML exhibited significantly lower potency. Notably, in intracellular amastigotes, MOX remained the most potent compound, while AFO also demonstrated similar efficacy followed by IVM. In contrast, PER and ML were significantly less effective, further highlighting their lower intracellular activity (Table 1). Cytotoxicity analysis in THP-1 cells revealed that PER displayed the highest cytotoxicity, whereas all other compounds exhibited significantly lower toxicity (Table 1). Consequently, SI analysis revealed that MOX had the highest selectivity, significantly greater than IVM, PER, and ML. AFO showed high selectivity, with no significant difference from either MOX or IVM. Contrarily, PER and ML had the lowest SI values, with no significant difference between them, indicating reduced selectivity (Table 1). To further assess the relevance of the observed differences in selectivity between the most selective compounds MOX and AFO, effect size analysis and Bayesian inference were employed. Cohen’s *d* effect size analysis (*d* = 1.79) demonstrated a large effect, indicating a substantial and meaningful difference favoring MOX over AFO, beyond statistical significance alone. Additionally, Bayesian analysis yielded a Bayes Factor (BF_10_ = 0.089), providing strong evidence in favor of MOX being superior to AFO. These findings highlight MOX as the most potent and selective compound, demonstrating superior efficacy across all *L. tropica* developmental stages while maintaining minimal cytotoxicity. Although AFO also exhibited high selectivity, the superiority of MOX was confirmed through additional statistical analyses, supporting its selection for further downstream investigations and preclinical evaluation.

**Table 1:** Efficacy of antiparasitic drugs against all developmental stages of *L. tropica*.

	Ivermectin	Permethrin	Moxidectin	Afoxolaner	Miltefosine	*p*-value
	IC_50_ ± SE (μM)					
Promastigotes	0.69 ± 0.04^a#^	1.72 ± 0.01^b^	0.58 ± 0.01^a^	0.6 ± 0.01^a^	1.57 ± 0.03^c^	<0.05
Axenic amastigotes	0.64 ± 0.00^a^	1.05 ± 0.02^b^	0.52 ± 0.01^c^	0.63 ± 0.00^a^	1.1 ± 0.04^b^	<0.05
Intracellular amastigotes	1.32 ± 0.07^a^	2.12 ± 0.04^c^	0.94 ± 0.06^b^	1.13 ± 0.03^ab^	2.33 ± 0.06^c^	<0.05
	CC_50_ ± SE (μM)					
THP-1 cells	60.44 ± 0.99^a^	42.66 ± 0.58^b^	60.29 ± 0.39^a^	60.70 ± 1.06^a^	62.33 ± 0.79^a^	<0.05
	Selectivity Index*					
	45.67 ± 2.45^a^	20.07 ± 0.5^c^	63.49 ± 4.3^b^	53.29 ± 1.87^ab^	26.75 ± 0.79^c^	<0.05

# Values within the same row with different superscript letters are significantly different (*p* < 0.05) according to Tukey’s HSD test. *Selectivity Index was calculated as the ratio of CC_50_ (THP-1 cells) to IC_50_ (intracellular amastigotes).

### Resistance acquisition of promastigotes and intracellular amastigotes

The artificial selection process led to a gradual and modest development of resistance in both promastigotes and intracellular amastigotes of *L. tropica* when exposed to the repurposed drugs and the positive control (Table 2). Notably, the resistance observed for the repurposed drugs was significantly lower compared to the resistance developed against ML in both promastigotes (*p* < 0.05; *F* value =5.867; df = 4) and intracellular amastigotes (*p* < 0.05; *F* value = 21.881; df = 4). Among promastigotes, PER and ML exhibited the highest RR after 15 selection rounds (20.09 ± 0.24 and 20.60 ± 1.72, respectively), significantly higher than all other compounds. IVM reached an RR of 20.02 ± 1.81, which was not significantly different from PER and ML. Notably, MOX displayed the lowest RR (17.23 ± 0.64), which was significantly lower than PER and ML, suggesting a slower resistance build-up despite prolonged selection pressure. AFO exhibited a comparable RR (17.28 ± 0.50), but MOX maintained the most controlled resistance progression among all tested compounds. A similar trend was observed in intracellular amastigotes, where RR values remained lower than in promastigotes, indicating a reduced resistance burden in the intracellular environment (Table 2). ML again displayed the highest RR after 15 selection rounds (7.21 ± 0.36), while MOX (4.59 ± 0.60) and AFO (4.91 ± 0.29) exhibited the lowest RR values (*p* < 0.05), reinforcing their superior resistance profiles. MOX, in particular, demonstrated the slowest resistance accumulation across all tested drugs, further highlighting its potential as a more sustainable therapeutic option. PER (5.72 ± 0.18) and IVM (4.70 ± 0.46) showed intermediate resistance levels, with IVM not significantly different from MOX and AFO, indicating a more moderate resistance trajectory (Table 2).

**Table 2:** Efficacy and resistance ratios (RR) for promastigotes and intracellular amastigotes of *L. tropica* under increasing drug pressure.

Generation	1		5		10		15	
	Promastigotes							
	IC_50_ ± SE (μM)	RR* ± SE	IC_50_ ± SE (μM)	RR ± SE	IC_50_ ± SE (μM)	RR ± SE	IC_50_ ± SE (μM)	RR ± SE
PER	1.72 ± 0.01	1.00 ± 0.00	4.25 ± 0.04	2.47 ± 0.04	15.58 ± 0.13	9.06 ± 0.12	34.56 ± 0.21	20.09^ab#^ ± 0.24
IVM	0.70 ± 0.08	1.00 ± 0.00	1.75 ± 0.01	2.52 ± 0.28	5.17 ± 0.09	7.46 ± 0.81	13.90 ± 0.34	20.02^ab^ ± 1.81
MOX	0.59 ± 0.03	1.00 ± 0.00	0.92 ± 0.01	1.56 ± 0.06	3.08 ± 0.07	5.24 ± 0.26	10.11 ± 0.08	17.23^a^ ± 0.64
AFO	0.60 ± 0.02	1.00 ± 0.00	0.95 ± 0.04	1.59 ± 0.10	3.38 ± 0.03	5.63 ± 0.16	10.36 ± 0.04	17.28^a^ ± 0.50
ML	1.51 ± 0.18	1.00 ± 0.00	3.52 ± 0.49	2.34 ± 0.22	13.68 ± 0.79	9.21 ± 1.66	30.83 ± 1.30	20.60^b^ ± 1.72
	Intracellular amastigotes							
	IC_50_ ± SE (μM)	RR* ± SE	IC_50_ ± SE (μM)	RR ± SE	IC_50_ ± SE (μM)	RR ± SE	IC_50_ ± SE (μM)	RR ± SE
PER	2.13 ± 0.08	1.00 ± 0.00	4.86 ± 0.04	2.29 ± 0.07	7.87 ± 0.08	3.71 ± 0.16	12.15 ± 0.06	5.72^b^ ± 0.18
IVM	1.32 ± 0.12	1.00 ± 0.00	2.22 ± 0.09	1.69 ± 0.21	4.35 ± 0.13	3.31 ± 0.37	6.18 ± 0.05	4.70^ab^ ± 0.46
MOX	0.95 ± 0.11	1.00 ± 0.00	1.24 ± 0.03	1.32 ± 0.16	3.07 ± 0.05	3.26 ± 0.40	4.31 ± 0.06	4.59^a^ ± 0.60
AFO	1.14 ± 0.06	1.00 ± 0.00	1.67 ± 0.02	1.47 ± 0.07	3.82 ± 0.02	3.36 ± 0.18	5.58 ± 0.04	4.91^ab^ ± 0.29
ML	2.34 ± 0.11	1.00 ± 0.00	3.14 ± 0.02	1.34 ± 0.06	6.86 ± 0.12	2.94 ± 0.10	16.83 ± 0.10	7.21^b^ ± 0.36

^**#**^Values within the same column with different superscript letters are significantly different (*p* < 0.05) according to Tukey’s HSD test. *Resistance ratios (RRs) were calculated by dividing the IC_50_ value of the generation under investigation by the IC_50_ value of the parental strain (first generation).

### Identification of differentially expressed genes by RNA-Seq following promastigotes and intracellular amastigotes exposure to MOX

**Figure 1:**
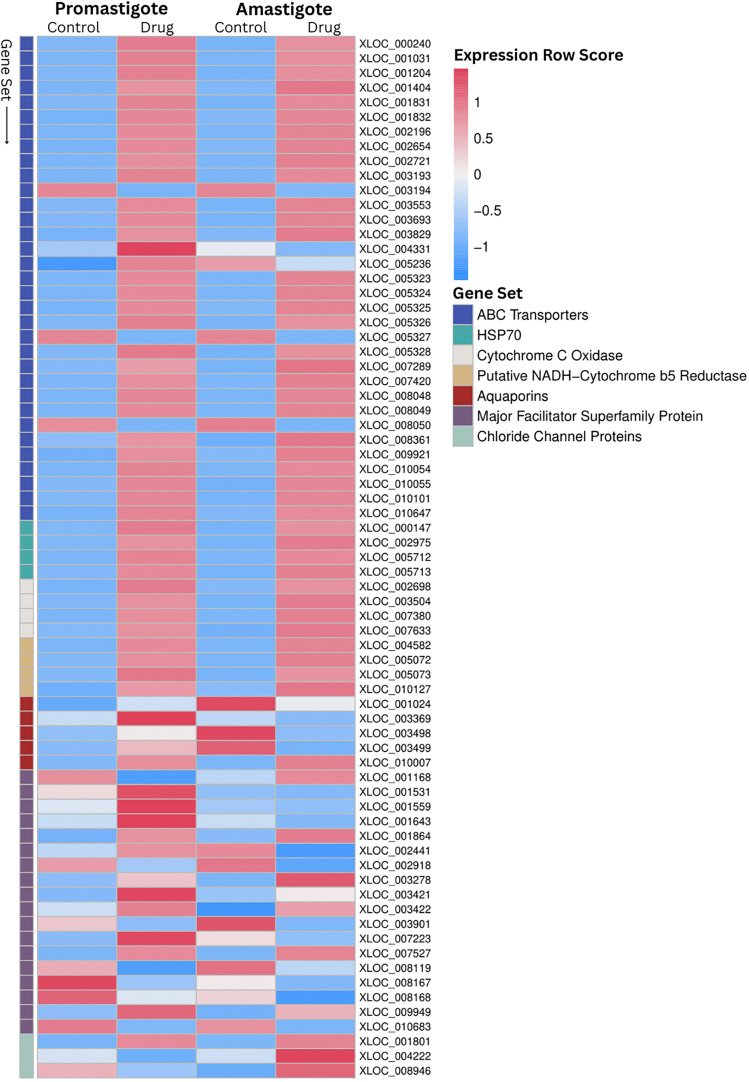
Heatmap of differentially expressed genes in *Leishmania tropica* promastigotes and amastigotes based on RNA-Seq expression values (Control *vs*. moxidectin-treated). The heatmap displays the normalized gene expression profiles derived from RNA-Seq differential expression analysis, comparing control and MOX-treated samples across promastigotes and amastigotes. Expression values are row *Z*-score normalized to highlight relative changes across samples with condition-specific patterns reflecting MOX-induced transcriptomic shifts.

**Supplementary Figure 1:**
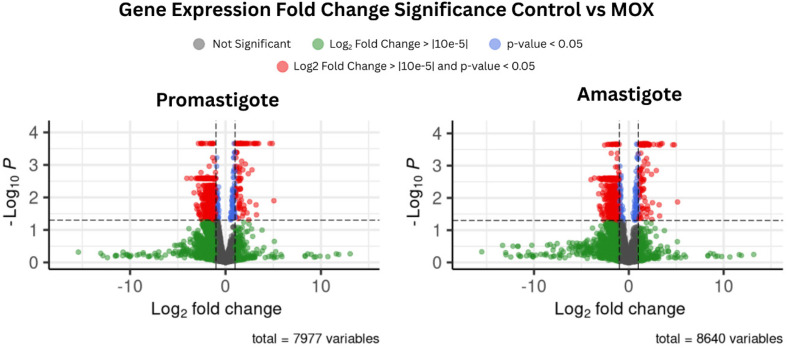
Differential gene expression profiles in *L. tropica* promastigotes and amastigotes following moxidectin (MOX) exposure.

**Supplementary Figure 2:**
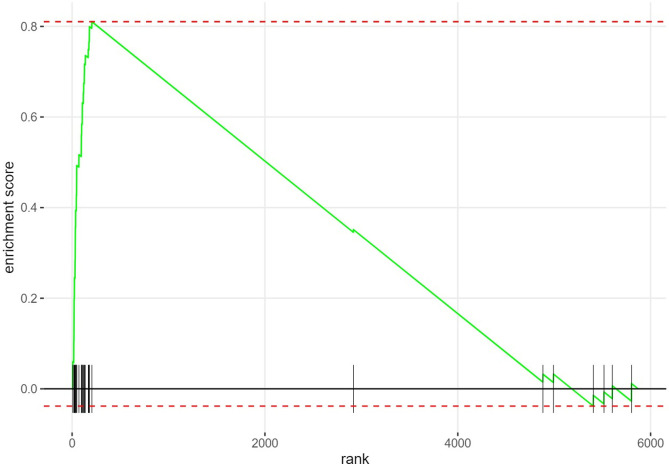
Gene set enrichment analysis (GSEA) plots for ABC transporters in intracellular amastigotes of *L. tropica*.

**Supplementary Figure 3:**
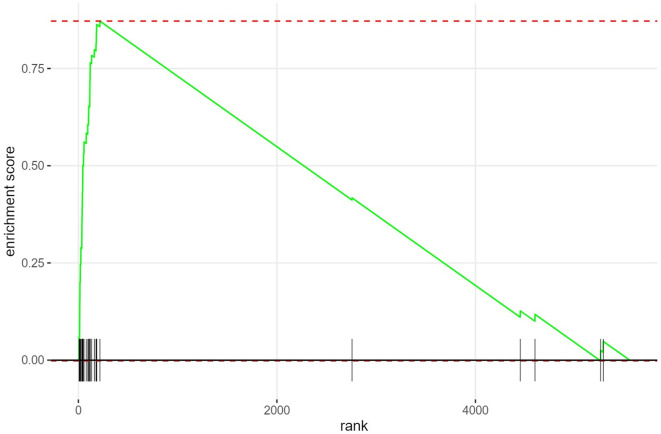
Gene set enrichment analysis (GSEA) plots for ABC transporters in promastigotes of *L. tropica*.

**Supplementary Figure 4:**
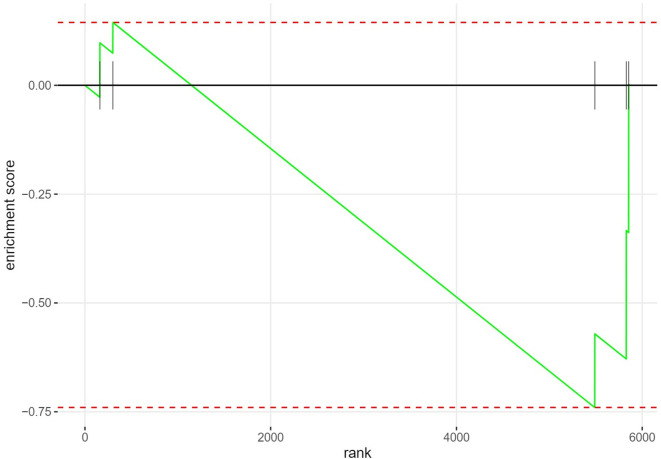
Gene set enrichment analysis (GSEA) plots for aquaporins in intracellular amastigotes of *L. tropica*.

**Supplementary Figure 5:**
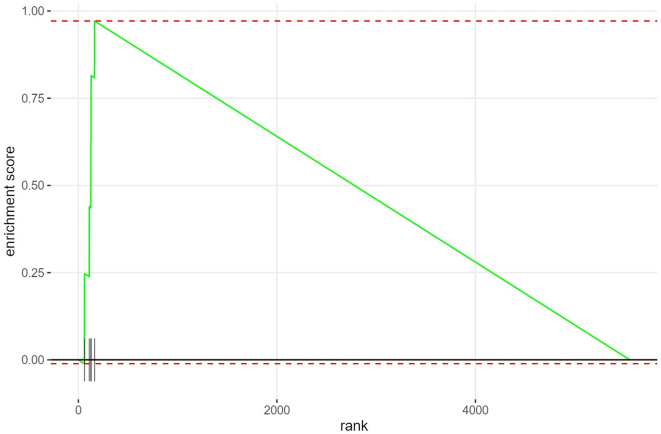
Gene set enrichment analysis (GSEA) plots for aquaporins in promastigotes of *L. tropica*.

**Supplementary Figure 8:**
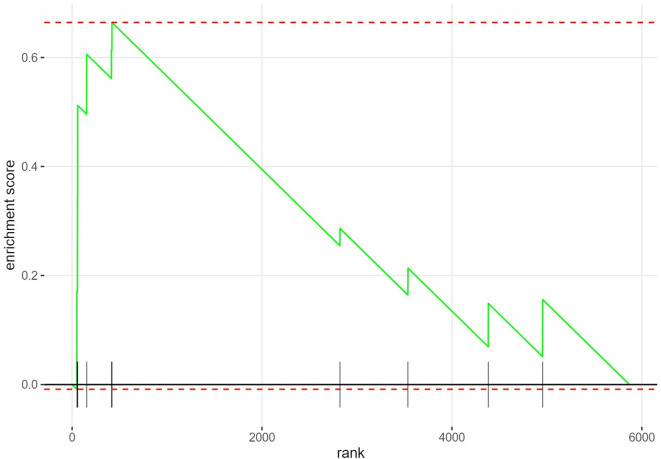
Gene set enrichment analysis (GSEA) plots for cytochrome c oxidase in intracellular amastigotes of *L. tropica*.

**Supplementary Figure 9:**
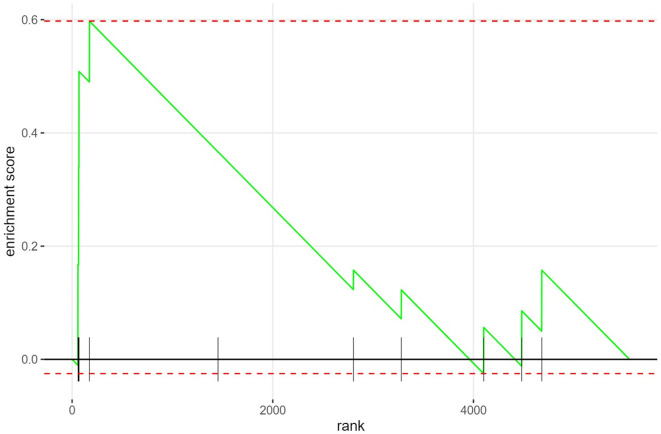
Gene set enrichment analysis (GSEA) plots for cytochrome c oxidase in promastigotes of *L. tropica*.

**Supplementary Figure 10:**
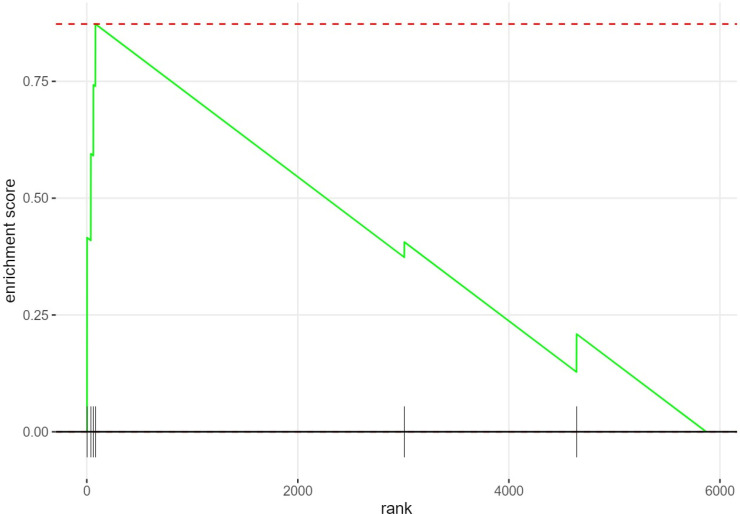
Gene set enrichment analysis (GSEA) plots for HSP70 in intracellular amastigotes of *L. tropica*.

**Supplementary Figure 11:**
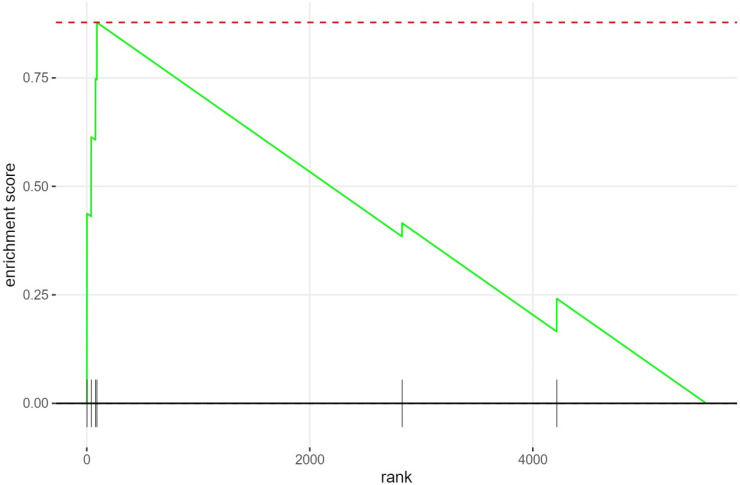
Gene set enrichment analysis (GSEA) plots for HSP70 in promastigotes of *L. tropica*.

**Supplementary Figure 12:**
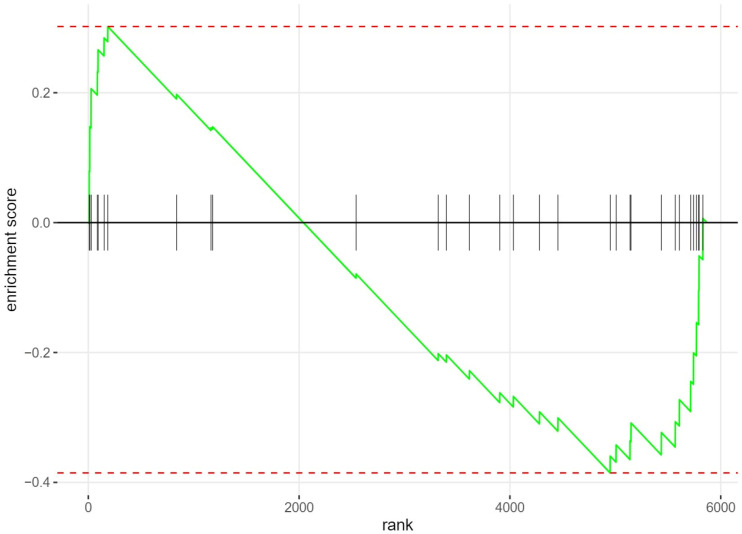
Gene set enrichment analysis (GSEA) plots for major facilitator superfamily (MFS) proteins in intracellular amastigotes of *L. tropica*.

**Supplementary Figure 13:**
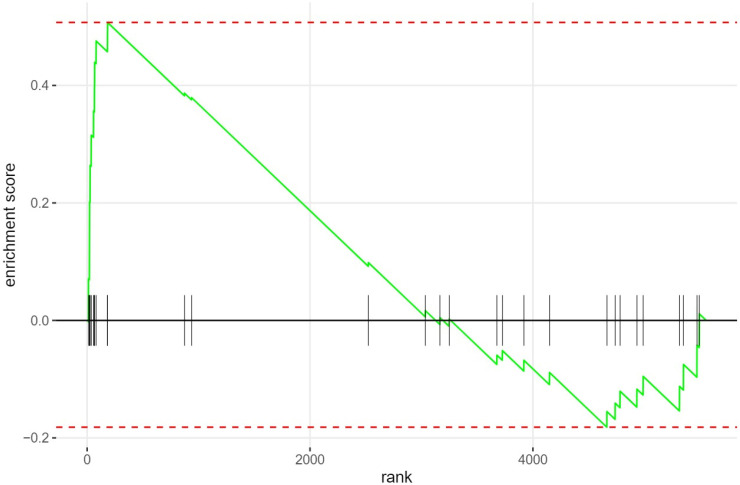
Gene set enrichment analysis (GSEA) plots for major facilitator superfamily (MFS) proteins in promastigotes of *L. tropica*.

**Supplementary Figure 14:**
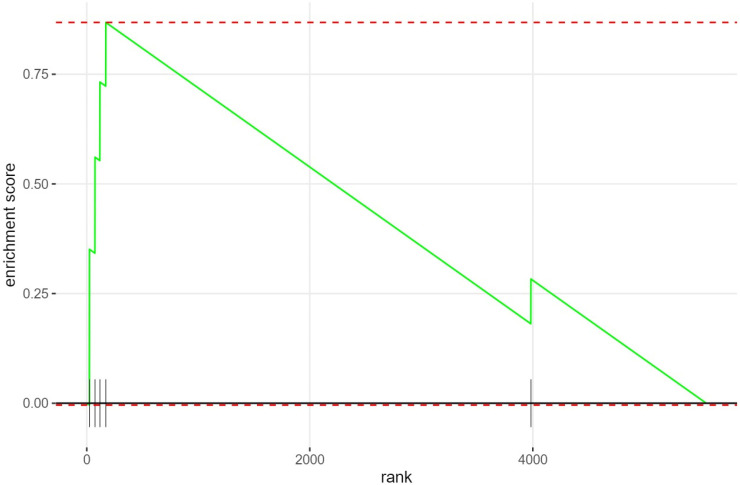
Gene set enrichment analysis (GSEA) plots for NADH-cytochrome b5 reductase in intracellular amastigotes of *L. tropica*.

**Supplementary Figure 15:**
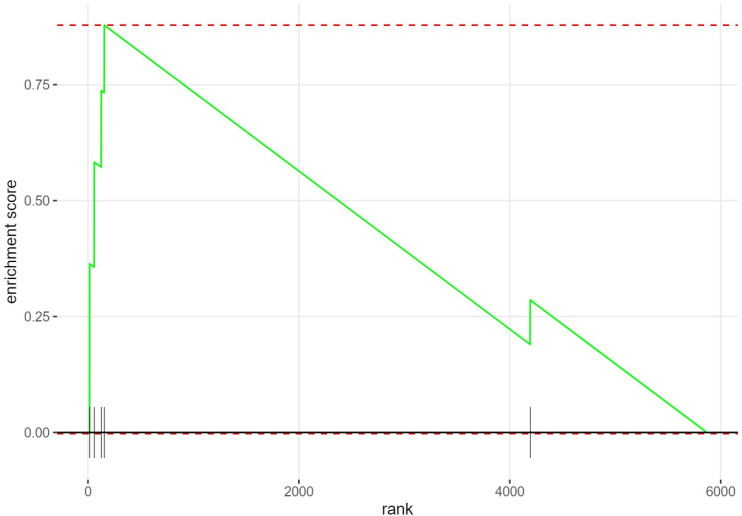
Gene set enrichment analysis (GSEA) plots for NADH-cytochrome b5 reductase in promastigotes of *L. tropica*.

All stages of *L. tropica* displayed high sensitivity and a minimal tendency to develop resistance to MOX, making it a promising candidate for further study. RNA-Seq was used to investigate MOX’s mode of action by analyzing changes in gene expression in promastigotes and intracellular amastigotes. In promastigotes, 812 out of 8,653 annotated genes (9.3%) were differentially expressed following MOX exposure, with 171 (21%) upregulated and 641 (79%) downregulated. Similarly, in intracellular amastigotes, 809 genes (9.3%) showed differential expression, with 166 (20.5%) upregulated and 643 (79.5%) downregulated (Supplementary Figure S1). Among the most significantly upregulated genes, the ABC transporter family gene, XLOC_007420, exhibited a fivefold increase in expression in both promastigotes and intracellular amastigotes (Table 3; Supplementary Tables S1 and S2). In contrast, the hypothetical protein XLOC_009739 showed an approximate fourfold decrease in expression in both stages. Gene set enrichment analysis revealed that the ABC transporter gene set, associated with drug resistance, was upregulated in both developmental stages (Table 3, Figure 1; Supplementary Figures S2 and S3). However, the major facilitator superfamily (MFS) gene set was upregulated only in promastigotes (Table 3, Figure 1; Supplementary Figures S4 and S5). The heat shock protein 70 (HSP70) gene set, which plays a role in protein folding, was upregulated in both stages (Figures 4 and 5). Additionally, gene sets related to parasite survival, including cytochrome c oxidase and NADH-cytochrome b5 reductase (Ncb5or), were upregulated in both promastigotes and intracellular amastigotes (Supplementary Figures S8–S11). Interestingly, the aquaporin gene set, involved in water and solute transfer, was upregulated in promastigotes but not in intracellular amastigotes (Supplementary Figures S12 and S13). Conversely, chloride channel (CLC) genes were downregulated in promastigotes but upregulated in intracellular amastigotes (Supplementary Figures S14 and S15). Furthermore, 227 putative genes coding for various proteins were associated with the differentially expressed genes, indicating broad molecular changes in response to MOX treatment.

**Table 3:** Representation of the differentially expressed gene sets in promastigotes and intracellular amastigotes with their GO IDs, the number of genes within the gene set, the number of differentially expressed genes within each gene set, and their functions (*p* < 0.05) following MOX exposure.

Gene set	GO ID	Function	Number of genes per gene set	Promastigotes					Intracellular amastigotes				
				Number of DEG	Expression level	*p*-value	Log2FC	ES	Number of DEG	Expression level	*p*-value	Log2FC	ES
ABC transporters	GO:0055085; GO:0005524; GO:0140359; GO:0016020	Transmembrane transport; ATP binding; ABC-type transporter activity; Membrane	46	35	Upregulated	6.973e-16	1.037	0.871	35	Upregulated	7.853e-15	0.986	0.81
HSP70	GO:0006457; GO:0005524; GO:0140662	Protein folding; ATP binding; ATP-dependent protein folding chaperone	8	6	Upregulated	0.003	0.431	0.877	6	Upregulated	0.0005	0.477	0.872
Cytochrome c oxidase	GO:0016491; GO:0016020	Oxidoreductase activity; membrane	11	10	Upregulated	0.037	0.321	0.597	10	Upregulated	4.455e-03	0.407	0.664
Putative NADH-cytochrome b5 reductase	GO:0004128	Cytochrome-b5 reductase activity, acting on NAD(P)H	6	5	Upregulated	0.01	0.38	0.868	5	Upregulated	0.002	0.431	0.878
Aquaporins	GO:0055085; GO:0015267; GO:0016020	Transmembrane transport; channel activity; membrane	5	5	Upregulated	0.0001	0.518	0.971	5	–	0.055	0.224	−0.74
Major Facilitator Superfamily protein	GO:0055085; GO:0022857; GO:0016020	Transmembrane transport; transmembrane transporter activity; membrane	33	30	Upregulated	0.001	0.455	0.507	31	–	0.357	0.066	−0.385
Chloride channel protein	GO:0006629; GO:0009086; GO:1902476; GO:0005247; GO:0016020	Lipid metabolic process; methionine biosynthetic process; chloride transmembrane transport; voltage-gated chloride channel activity; membrane	3	3	Downregulated	0.001	0.455	−0.982	3	Upregulated	0.001	0.455	0.97

### Measurement of chloride ions in promastigotes of *Leishmania tropica*

To elucidate the modulatory effect of MOX on CLCs, we conducted a comprehensive analysis of chloride concentration in promastigotes of *L. tropica*. In control promastigotes (no drug exposure), the baseline chloride concentration was determined to be 141.02 ± 4.04 nmol/mL. Remarkably, this concentration exhibited a significant decrease (97.33 ± 6.02 nmol/mL) and increase (337.67 ± 26 nmol/mL) upon the introduction of A9C and MOX, respectively (*p* < 0.05; *F* value = 193.677; df = 2).

### Molecular docking and dynamic simulation

**Figure 2:**
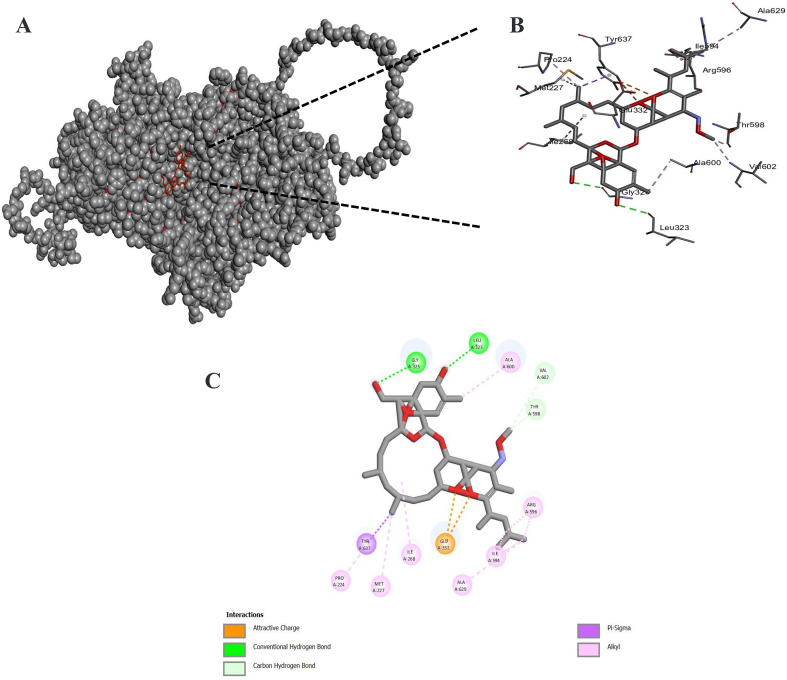
Molecular docking of MOX with *Leishmania tropica* chloride channel: (A) surface representation of protein in grey and MOX is shown in red and (B) three-dimensional (2D) diagram of interacting residues. (C) 2D diagram of interacting residues.

We employed template-based modeling to predict the structure of the *L. tropica* CLC, with the model quality assessed using the global model quality estimate (GMQE) metric, which scored 0.78, indicating a robust and reliable model. Further validation through a Ramachandran plot confirmed that 91.3% of residues were in favorable conformations, supporting the model’s accuracy. Molecular docking analysis revealed that MOX exhibited the lowest binding affinity with the CLC selectivity filter compared to control ligands (Table 4). The interaction profile indicated that MOX primarily interacted with the CLC through van der Waals forces (Val602), hydrogen bonds (Gly352, Leu323, and Thr598), and alkyl interactions (Pro224, Met227, Ile268, Ala629, Arg596, and Ala600), with additional π–σ interactions involving Tyr637 (Figure 2). MOX exhibited the highest binding affinity to the selectivity filter of the CLC, a specialized structural motif responsible for the selective permeation of chloride ions while excluding other ions based on size and charge [42].

**Table 4:** Binding affinity of moxidectin and different known modulators to the chloride channel of *Leishmania tropica*.

	Binding energy (kcal/mol)		
Domain	Moxidectin	9-anthracene-carboxylic acid (9-AC)	Lubiprostone
Selectivity filter	−9.24	−4.75	−3.21
Ligand binding site I	−2.18	−1.08	−4.37
Ligand binding site II	−1.22	−2.43	−3.48

**Figure 3:**
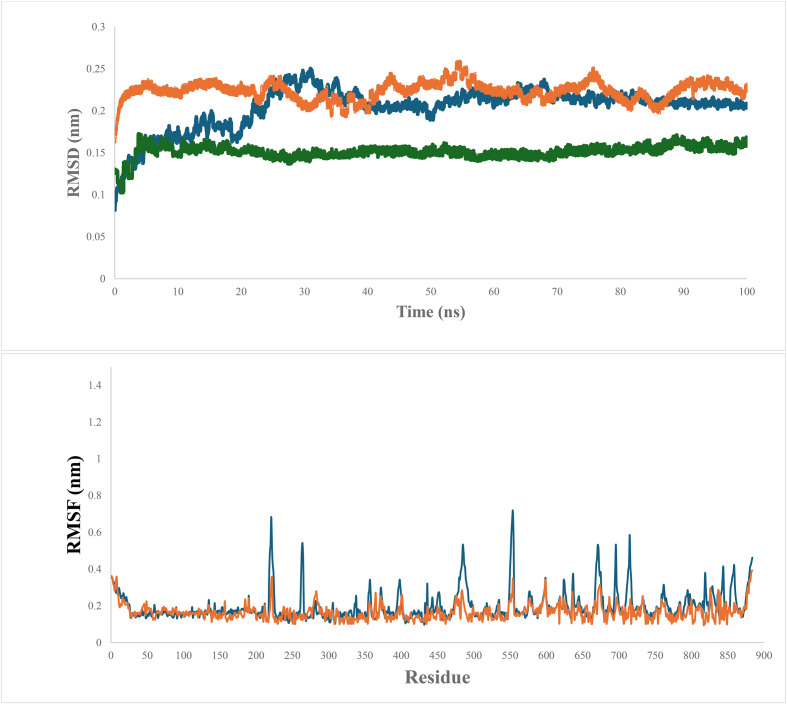
(A) Root mean square deviation (RMSD) of the chloride channel of *Leishmania tropica* in complex with MOX, during 100 ns of the molecular dynamic (MD) simulation period. (B) Root mean square fluctuation (RMSF) of the chloride channel of *Leishmania tropica* in complex with MOX, during 100 ns of the MD simulation period.

Subsequent molecular dynamics (MD) simulations evaluated the stability of the MOX-CLC interaction. The RMSD values for the complex (ligand-bound protein), the unbound protein, and the ligand were 0.22 nm, 0.2 nm, and 0.15 nm, respectively indicating stable binding throughout the simulation (Figure 3). Root mean square fluctuation (RMSF) analysis showed heightened fluctuations in amino acids near the selectivity filter, such as Gly220, Gly222, Gly262, Gly265, Gly555, Ser221, Ile223, Ile554, Pro224, Pro266, Pro555, Thr263, The552, Glu264, Val553, and Tyr637, suggesting localized structural dynamics around the binding site. Notably, the highly conserved Glu264 residue, located near the extracellular entrance of the pore, showed prominent fluctuations. The side chain of this glutamate residue projects into the lumen of the channel, enabling direct interaction with positively charged ions passing through [47]. This electrostatic interaction acts as a barrier to negatively charged chloride ions, influencing the channel’s conductance. The critical role of this conserved glutamate residue has been demonstrated through site-directed mutagenesis experiments, where substitution with other amino acids abolishes voltage- and chloride-dependent gating in CLCs, often leading to significant alterations in the channel’s conductance, gating properties, and pH sensitivity [42].

Minimal fluctuations were observed in other regions, indicating the overall stability of the MOX-CLC complex. The MM/PBSA method was used to calculate binding free energy, with MOX showing a favorable binding energy of −123.7 kJ/mol (Supplementary Table S3). Van der Waals interactions were the dominant contributors to the low binding energy, indicating strong and stable MOX-CLC binding (Supplementary Table S3).

### *In vivo* efficacy of MOX against leishmaniasis using *Galleria mellonella* as an infection model

**Figure 4:**
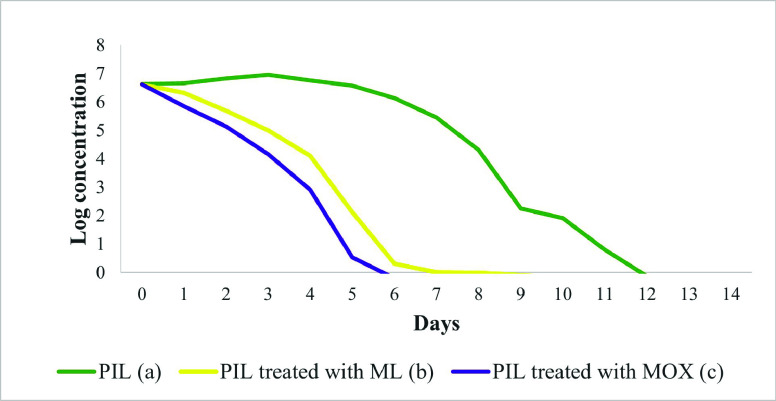
*In vivo* time-dependent reduction in *Leishmania tropica* load following treatment with miltefosine (ML) and moxidectin (MOX) using *Galleria mellonella* as the infection model. Statistical analysis of the area under the curve (AUC) revealed significant differences between all groups (*p* < 0.05; one-way ANOVA with Tukey’s HSD test). Groups with different letters are significantly different: PIL (a) 57.37 (SE = 0.19); PIL treated with ML (b) 25.60 (SE = 0.30); PIL treated with MOX (c) 19.32 (SE = 0.24).

**Figure 5:**
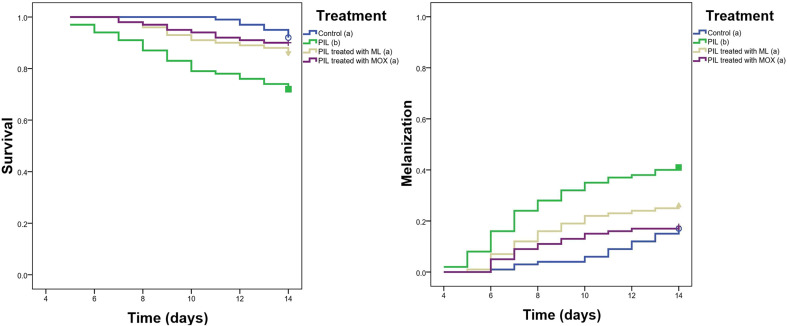
Kaplan-Meier survival and melanization curves for *Galleria mellonella* larvae infected with *Leishmania tropica* and treated with miltefosine (ML) or moxidectin (MOX). Statistical comparisons were performed using the log-rank (Mantel-Cox) test. Chi-square (χ^2^) values reflect the magnitude of divergence between groups over time. Groups with different letters are significantly different. For mortality: PIL (b) (χ^2^ = 14.773); PIL + ML (a) (χ^2^ = 2.073); PIL + MOX (a) (χ^2^ = 0.335). For melanization: PIL (b) (χ^2^ = 16.591); PIL + ML (a) (χ^2^ = 3.051), PIL + MOX (a) (χ^2^ = 0.114). Significant differences were observed between PIL and treated groups (*p* < 0.05), while no significant differences were detected between treated and control groups (*p* > 0.05).

The *in vivo* therapeutic efficacy of MOX was evaluated using a *Galleria mellonella* insect model over a 14-day period. RNA was extracted from the hemocoel of *G. mellonella* infected with *L. tropica* and analyzed using RT-qPCR, showing a significant reduction in the *L. tropica* burden in larvae treated with MOX (Figure 4). The AUC was calculated for each larva to quantify the overall therapeutic effect. Mean AUC values were 57.37 (SE = 0.19) for promastigote-infected larvae (PIL), 25.60 (SE = 0.30) for PIL treated with ML, and 19.32 (SE = 0.24) for PIL treated with MOX. A one-way ANOVA revealed a highly significant difference in AUC between the groups (*F*(2, 57) = 6,84,109.55, *p* < 0.05). Tukey’s honest significant difference (HSD) test indicated statistically significant differences between all pairs: PIL *vs*. PIL treated with ML (mean difference = 31.77, *p* < 0.05), PIL *vs*. PIL treated with MOX (mean difference = 38.05, *p* < 0.05), and PIL treated with ML *vs*. PIL treated with MOX (mean difference = 6.28, *p* < 0.05). These results demonstrate that MOX exhibited the highest therapeutic efficacy, with the greatest reduction in *L. tropica* burden. Mortality and melanization rates in the control group remained below 5% during the first 13 and 10 days post-exposure, respectively. Figure 5 displays the survival and melanization curves for each experimental group. Significantly higher mortality and melanization rates were observed in PIL compared to the control group, while no significant differences were detected between the control group and larvae treated with MOX or ML, indicating effective therapeutic outcomes for both treatments. Based on the log-rank test results derived from Kaplan-Meier survival and melanization analyses, the groups ranked as follows: PIL (χ^2^ = 14.773; df = 1; *p* < 0.05) > PIL treated with ML (χ^2^ = 2.073; df = 1; *p* > 0.05) > PIL treated with MOX (χ^2^ = 0.335; df = 1; *p* > 0.05) for mortality, and PIL (χ^2^ = 16.591; df = 1; *p* < 0.05) > PIL treated with ML (χ^2^ = 3.051; df = 1; *p* > 0.05) > PIL treated with MOX (χ^2^ = 0.114; df = 1; *p* > 0.05) for melanization. These findings confirm that MOX provided the most effective treatment against *L. tropica* in the *G. mellonella* model.

## Discussion

Leishmaniasis is a vector-borne disease transmitted to humans through the bite of an infected female sandfly [37, 65]. The lack of effective therapeutic interventions has contributed to its persistent global impact, affecting millions [60]. Among parasitic diseases, leishmaniasis ranks alongside malaria, filariasis, tuberculosis, and leprosy in terms of health burden, leading to its classification as a category 1 disease [12, 62]. Current treatments, including pentavalent antimonials, miltefosine (ML), parenteral paromomycin, and amphotericin B [57], are associated with significant drawbacks such as high toxicity, cost, relapse risk, and the development of drug resistance [60]. Unfortunately, the development of new therapeutic options remains limited due to insufficient investment in time and financial resources, with only a few potential drugs in the pipeline [60]. A pivotal strategy in addressing the treatment of leishmaniasis is therapeutic switching, which offers distinct advantages over conventional drug discovery [58]. Our study is the first to evaluate the efficacy of antiparasitic agents – PER, IVM, MOX, and AFO – across all developmental stages of *L. tropica* as well as THP-1 cells. With the exception of PER, all repurposed drugs demonstrated a relatively high selectivity index, compared to ML. A dose-dependent potency was shown against all parasite stages with minimal cytotoxic effects on THP-1 cells (Table 1). IVM, a macrocyclic lactone with broad antiparasitic activity, has proven effective against various parasitic infestations [39]. Its efficacy also extends to *L. major* [59] and *L. amazonensis* [48], with analogs like Δ2,3-IVM exhibiting a high selectivity index and minimal impact on mammalian ATPases at effective concentrations. In contrast to previous studies, our findings indicate different effective concentrations, likely due to species-specific susceptibility variations among *Leishmania* species [48, 64]. Strikingly, MOX, a macrocyclic lactone belonging to the milbemycin subclass and closely related to IVM, showed even greater efficacy against all developmental stages of *L. tropica* (Table 1). Despite their shared mechanisms of action, MOX and IVM exhibit distinct pharmacokinetic profiles. MOX has demonstrated increased bioavailability, a prolonged half-life in mammalian cells, and a broader spectrum of antiparasitic activity [7, 18], which may contribute to its superior performance. The roles of pyrethroids (PER) and isoxazolines (AFO) in treating *Leishmania spp.* are not well-documented. Studies have shown that PER-treated bed nets reduce sandfly populations and leishmaniasis transmission [61], while PER-treated collars protect dogs against sandfly bites, lowering canine visceral leishmaniasis incidence [23]. Similarly, oral AFO administration in dogs eradicates sandflies [54]. Historically, these drugs have primarily been used in preventive strategies by reducing vector contact and sandfly density. However, our study reveals their potential as curative agents, suggesting a new therapeutic role for these compounds against *L. tropica*. Notably, the efficacy of these drugs varied across different developmental stages of the parasite, with the highest susceptibility observed in axenic amastigotes, followed by promastigotes, and then intracellular amastigotes (Table 1). This stage-dependent efficacy introduces a novel dimension to their potential application in treating leishmaniasis. The susceptibility of the parasite forms follows a distinct sequence: axenic amastigotes are the most susceptible, followed by promastigotes, with intracellular amastigotes being the least susceptible. This aligns with findings that reported that axenic amastigotes exhibit greater vulnerability to drug exposure than promastigotes [76]. The observed chemoresistance in intracellular amastigotes compared to their flagellated counterparts (Table 2) appears to be linked to the reduced ability of molecules to penetrate the host cell membrane, a result of the unique intracellular environment. While efforts to evaluate new anti-leishmanial therapeutics are ongoing, researchers face the challenge of proactively assessing potential drug resistance [57]. *Leishmania* spp. demonstrate remarkable adaptability and a rapid ability to acclimate to environmental pressures, often developing resistance to xenobiotics [44]. Given the significant threat posed by resistance acquisition, which can compromise drug efficacy, we investigated resistance development in both promastigotes and intracellular amastigotes of *L. tropica* (Table 2). Traditionally, resistance studies have focused on promastigotes due to the ease of culturing and applying selection pressure [23]. However, this approach has a critical limitation, as resistance acquired in one developmental stage may not directly translate to another [28]. Hendrickx and colleagues emphasized that promastigotes might not be the most appropriate model for studying resistance acquisition since intracellular amastigotes are the most clinically relevant stage [28]. In our study, the RR values for ML showed significant increases, with a 20.6-fold rise in promastigotes and a 7.21-fold rise in intracellular amastigotes (Figure 1). These increases were notably higher than those observed with repurposed drugs, underscoring the rapid emergence of ML resistance compared to other treatments (Table 2). This trend aligns with numerous previous studies demonstrating the rapid emergence of ML resistance in *Leishmania* spp*.* [23, 29, 52]. The development of drug resistance is often influenced by pharmacokinetic factors [41]. In the case of ML, its relatively slow elimination rate results in prolonged suboptimal drug levels within patients, which can reduce its therapeutic efficacy and contribute to resistance [20]. However, caution is warranted when interpreting the observed higher resistance acquisition to ML compared to the repurposed drugs. Instances of resistance to the original indications of these repurposed drugs have been documented [26, 35]. Therefore, it is conceivable that *L. tropica* could develop resistance to these repurposed drugs over time, potentially leading to treatment failures in managing leishmaniasis in the future.

Given the high potency and selectivity index demonstrated by MOX against *L. tropica*, further investigations were prioritized to elucidate its mechanism of action. While AFO also exhibited strong efficacy, MOX’s superiority supports its selection for downstream investigations. Understanding MOX’s molecular targets and cellular impact is essential for assessing its therapeutic potential and differentiating its mode of action from other macrocyclic lactones. The mechanism of action for macrocyclic lactones, such as MOX, is primarily linked to the activation of gamma-aminobutyric and glutamate-gated CLCs in parasite nervous systems, leading to chloride influx, hyperpolarization, and paralysis [66]. However, since *Leishmania* lacks a conventional nervous system, the impact of MOX on this protozoan necessitates more nuanced exploration. Chloride ions play crucial roles in *Leishmania*’s cellular processes, including osmoregulation, pH homeostasis, cellular signaling, and electrochemical balance [27]. Disruptions in chloride levels can lead to cellular stress or impaired signaling, highlighting the importance of maintaining intracellular osmotic balance through chloride transport channels [27]. Our RNA-Seq analysis revealed that MOX targets CLCs in both promastigotes and intracellular amastigotes, with downregulation of three CLC genes and two ion-selective channel genes in promastigotes, but upregulation in intracellular amastigotes (Table 3, Figure 1; Supplementary Table S4). This suggests that MOX disrupts intracellular osmotic balance through CLC neutralization. In nutrient-rich environments like the RPMI medium, MOX may induce significant chloride influx in promastigotes, causing downregulation of chloride channels. This mirrors findings in leukemia cells, where MOX increased intracellular chloride concentration, resulting in cell death [46]. Conversely, in nutrient-poor environments like the phagolysosome, neutralization of transport channels by MOX likely causes ion efflux, prompting intracellular amastigotes to upregulate these channels to counteract the imbalance [67]. Another notable observation is the upregulation of aquaporin genes in promastigotes following MOX exposure (Table 3, Figure 1; Supplementary Table S4). Disruption of CLCs by MOX leads to ion imbalances, triggering osmotic stress in promastigotes. Aquaporins, which regulate water and solute movement across cell membranes, may be upregulated as a compensatory mechanism, as seen in studies demonstrating their role in metabolite release, nutrient uptake, and stress alleviation in protozoan parasites [70]. Interestingly, no differential expression of aquaporins was observed in intracellular amastigotes, possibly due to the nutrient-poor conditions within the phagolysosome affecting cellular signaling pathways differently, suggesting stage-specific responses in *Leishmania*. The major facilitator superfamily (MFS) proteins, which mediate the transport of ions, amino acids, and other molecules, were also affected by MOX exposure. Our data indicated overexpression of MFS genes in promastigotes, likely due to elevated iron levels, suggesting a role for the Leishmania Iron Regulator 1 (LIR1) protein in controlling intracellular iron concentration [40, 77]. Intriguingly, this response was absent in intracellular amastigotes, potentially due to macrophages restricting their access to iron [31]. Studies have shown that other transporters, such as transferrin receptors, may compensate for this iron scarcity [24]. Our RNA-Seq analysis further identified overexpression of the ATP-binding cassette (ABC) transporter family in both stages of *L. tropica* following MOX exposure (Table 3, Figure 1; Supplementary Table S4). ABC transporters are known for their role in drug resistance, using ATP hydrolysis to transport xenobiotic compounds across membranes, thereby reducing drug absorption and bioavailability [3]. This stage-independent overexpression may reflect an immune-like response against a variety of drugs, consistent with findings showing that ABC transporters in *L. tropica* have a strong affinity for ML, reducing its intracellular accumulation [53]. Moreover, eight distinct HSP70 genes were upregulated in response to MOX exposure (Table 3, Figure 1; Supplementary Table S4). Heat shock proteins (HSPs) prevent protein aggregation and play a critical role in stress tolerance and drug resistance [55]. Previous studies have demonstrated that overexpression of HSPs correlates with increased drug resistance in *Leishmania*, particularly in response to pentavalent antimony, methotrexate, nelfinavir, and amphotericin B [14, 16]. Lastly, MOX exposure led to the upregulation of ten genes encoding cytochrome c oxidase (CcO) in both stages, suggesting a potential impact on mitochondrial respiratory chain activity (Table 3, Figure 1; Supplementary Table S4). CcO serves as the terminal oxidase in this complex, and disruptions in mitochondrial function can impact numerous biological processes [19]. Previous studies have shown that ML inhibits CcO in *L. donovani*, and a similar effect may be occurring with MOX [42]. This upregulation likely represents a cellular response to compensate for reduced CcO activity, attempting to restore energy generation. The observed upregulation of Ncb5or genes following MOX exposure also sheds light on potential effects on *L. tropica*’s lipid metabolism. Ncb5or catalyzes the conversion of oleate to linoleate, a lipid that deactivates the NF-κB signaling pathway in macrophages [47]. Enhanced lipid metabolism may aid *Leishmania* in adapting to MOX-induced stress, aligning with studies showing that Ncb5or plays a role in inhibiting oxygen consumption, ROS, and ATP production, thereby preventing apoptosis [47].

While RNA-Seq serves as a comprehensive tool for analyzing drug mechanisms, identifying numerous differentially expressed genes might not fully capture the complexity of a drug’s action. The inherent intricacies of biological systems make it challenging to pinpoint all the molecular pathways influenced by a drug. MOX, initially recognized for its selective targeting of glutamate-gated CLCs in invertebrates, leading to increased chloride efflux, was found in this study to influence a broader spectrum of CLCs in *L. tropica*. This suggests that *Leishmania* CLCs may serve as a significant target in MOX’s therapeutic action, contributing to an ionic imbalance. Our understanding of CLCs within protozoan plasma membranes, especially in *Leishmania*, remains limited due to scarce information on their functional properties [34]. Despite their fundamental role in cellular processes, further research is required to elucidate the molecular mechanisms and regulatory pathways governing CLC function in protozoans [34]. Previous studies demonstrated the impact of CLC modulators on *Leishmania* developmental stages, but the comprehensive characterization of these channels is crucial for developing effective antileishmanial agents [56]. Our docking and MD simulations revealed that MOX stably binds to the CLC selectivity filter, particularly interacting with Glu264, a residue critical for ion selectivity. These findings strongly support our hypothesis that MOX binds to and neutralizes the selectivity filter of the CLC, potentially facilitating passive chloride ion diffusion down its concentration gradient.

It is crucial to acknowledge the limitations of our study, as we used a protein structure predicted by AlphaFold, and CLCs in *Leishmania* spp*.* have not undergone comprehensive characterization. Despite AlphaFold’s remarkable accuracy, potential structural inaccuracies remain. Therefore, our molecular dynamics simulations should be interpreted with caution, and these findings require validation through complementary experimental approaches. The integration of experimental data, such as cryo-electron microscopy densities or nuclear magnetic resonance restraints, could refine and validate the AlphaFold-predicted structure of the *L. tropica* CLC.

The *in vitro* anti-leishmanial activity of MOX against *L. tropica* observed in our cell culture assays provided a strong foundation for evaluating its efficacy in an *in vivo* model system. However, cellular assays alone cannot fully replicate the complex physiological environment and immune responses of living organisms. The *G. mellonella* larva has emerged as a valuable infection model for assessing the therapeutic potential of compounds against various pathogens, offering advantages such as a functional innate immune system, ease of drug administration, and ethical considerations over mammalian models [45, 51]. Notably, our results demonstrated a strong correlation between the *in vitro* and *in vivo* therapeutic efficacy of MOX, with the *G. mellonella* model validating the potent activity observed in cellular assays (Figure 4). The significant reduction in *Leishmania* cell counts, as well as reduced melanization and mortality (Figure 5), suggests that MOX exhibits favorable pharmacodynamics and pharmacokinetic properties, promoting effective distribution and accumulation at the infection site. MOX’s unique chemical structure, featuring a macrocyclic lactone ring system, may contribute to its antiparasitic potency. Unlike the first-generation macrocyclic lactones, MOX lacks a disaccharide moiety at carbon-13, which simplifies its structural configuration and may influence its pharmacokinetic attributes and metabolic stability [10]. Additionally, MOX’s olefinic side chain at carbon-25 may alter ligand-receptor interactions and membrane permeability, enhancing its antiparasitic efficacy [10]. The highly lipophilic core likely facilitates MOX’s enhanced permeability across biological membranes, enabling it to penetrate cellular barriers and accumulate within the phagolysosomes harboring intracellular amastigotes. These pharmacokinetic and chemical characteristics potentially explain MOX’s remarkable anti-leishmanial efficacy in our infection model, warranting further investigation in higher mammalian models and clinical studies. While the *G. mellonella* model offers an efficient initial platform for evaluating therapeutic potential [51], it is important to recognize its inherent limitations. Also, *G. mellonella* lacks a fully developed adaptive immune system, relying exclusively on innate immune responses [73]. Therefore, the anti-leishmanial activity of MOX observed in this model might not accurately reflect its efficacy in a mammalian system, where adaptive immunity plays a crucial role in the pathogenesis and resolution of leishmaniasis [17]. Moreover, the physiological processes governing drug absorption, distribution, metabolism, elimination, and toxicity in *G. mellonella* can differ significantly from those in mammalian systems, potentially leading to differences in pharmacokinetic profiles and tissue exposure. Additionally, the *G. mellonella* model does not fully mimic the diverse clinical manifestations and pathophysiological complexities of human leishmaniasis, such as granuloma formation, disseminated infections, and immunopathological responses. Therefore, while this insect model serves as a valuable preliminary screening tool, the anti-leishmanial efficacy of MOX observed here should be interpreted with caution. Validation in higher mammalian models, using varied treatment regimens, is crucial to accurately evaluate MOX’s therapeutic potential, pharmacokinetic behavior, and safety profile under conditions that more closely resemble human leishmaniasis.

## Conclusion

This study explored the potential of repurposing MOX, a macrocyclic lactone approved for veterinary use, as an antileishmanial agent targeting all developmental stages of *L. tropica*. Among the five antiparasitic drugs tested, MOX demonstrated the highest efficacy against both promastigotes and intracellular amastigotes with minimal resistance. RNA-Seq analysis revealed complex gene expression changes in response to MOX exposure, suggesting its disruption of ionic homeostasis via chloride channel CLC modulation, ultimately impairing parasite survival. MOX’s unique mechanism of action, distinct from existing antileishmanial drugs, may help overcome resistance, while offering a promising therapeutic option for veterinary and potentially human applications. Its established safety profile, widespread veterinary use, and well-documented pharmacokinetics could streamline development, reducing costs and expediting regulatory approval. While further studies are needed to determine optimal dosing, safety, and efficacy in both veterinary and human settings, MOX represents a viable, cost-effective alternative for leishmaniasis treatment, particularly in resource-limited regions where new therapeutic options are urgently needed.

## Figures and Tables

**Supplementary Figure 6:**
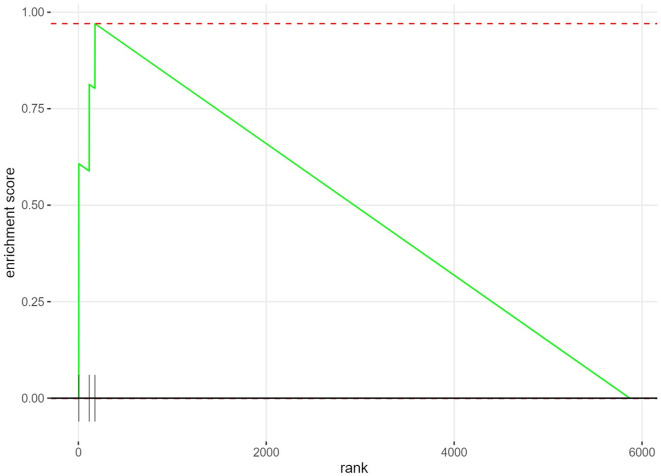
Gene set enrichment analysis (GSEA) plots for chloride channels in intracellular amastigotes of *L. tropica*.

**Supplementary Figure 7:**
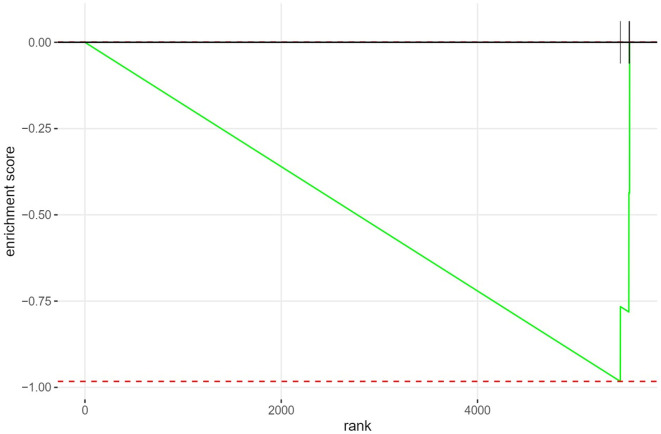
Gene set enrichment analysis (GSEA) plots for chloride channels in promastigotes of *L. tropica*.

## Supplementary material

The supplementary material for this article can be found at https://www.parasite-journal.org/10.1051/parasite/2025035/olm.
*Supplementary Note 1*: Functional characterization of differentially expressed gene families.
*Supplementary Table 1*: Average binding free energies calculated from molecular dynamics (MD) simulations for moxidectin (MOX) and the chloride channel of *Leishmania tropica*.
*Supplementary Table 2*: Top 10 upregulated and downregulated genes in *L. tropica* promastigotes following moxidectin (MOX) exposure, with corresponding log2 fold change values and statistical significance.
*Supplementary Table 3*: Top 10 upregulated and downregulated genes in *L. tropica* intracellular amastigotes following moxidectin (MOX) exposure, with corresponding log2 fold change values and statistical significance.
*Supplementary Table 4*: List of differentially expressed genes within the seven assembled gene sets, including ABC transporters, HSP70, cytochrome c oxidase, NADH-cytochrome b5 reductase, aquaporins, major facilitator superfamily (MFS), and chloride channels.
