# Supplementary material for: Repositioning of moxidectin: a promising approach in cutaneous leishmaniasis therapy
Source: Parasite. 2025 Jul 4;32:42. doi: 10.1051/parasite/2025035 (PMC12232414; doi:10.1051/parasite/2025035)
Supplement: Supplementary file 1 — Supplementary Note 1: Functional characterization of differentially expressed gene families. [file parasite-32-42-s1.pdf]

Gene families

Notably, the chloride channel gene set emerged as remarkable. This gene set comprised three genes encoding proteins associated with the following Gene Ontology (GO) terms: GO:0006629 (lipid metabolic process), GO:0009086 (methionine biosynthetic process), GO:1902476 (chloride transmembrane transport), GO:0005247 (chloride channel activity), and GO:0016020 (membrane) (Table 1). Furthermore, the aquaporin gene set, comprising five genes annotated with GO:0055085 (transmembrane transport), GO:0015267 (channel activity), and GO:0016020 (membrane), was implicated in mediating osmotic balance and regulation. The major facilitator superfamily (MFS) gene set, involved in facilitating iron efflux, encompassed 33 genes (Table 1) engaged in transmembrane transport, as defined by GO:0055085, GO:0022857 (transmembrane transporter activity), and GO:0016020. Notably, the ATP-binding cassette (ABC) transporter gene set, comprising 46 genes (Table 1) sharing GO:0055085, GO:0005524 (ATP binding), GO:0140359 (ABC-type transmembrane transporter activity), and GO:0016020, was of particular interest due to its potential role in drug resistance mechanisms. To mitigate cellular stress, the heat shock protein 70 (HSP70) gene set, comprising eight genes (Table 1) annotated with GO:0006457 (protein folding), GO:0005524, and GO:0140662 (ATP-dependent protein folding chaperone activity), was assembled. Parasite survival-related processes were represented by the cytochrome c oxidase gene set, consisting of 11 genes (Table 1) associated with GO:0016491 (oxidoreductase activity) and GO:0016020. Additionally, the NADH-cytochrome b5 reductase (Ncb5or) gene set, comprising six genes (Table 1) annotated with GO:0004128 (cytochrome-b5 reductase activity), was implicated in enhanced lipid metabolism.
