# Supplementary material for: Repositioning of moxidectin: a promising approach in cutaneous leishmaniasis therapy
Source: Parasite. 2025 Jul 4;32:42. doi: 10.1051/parasite/2025035 (PMC12232414; doi:10.1051/parasite/2025035)
Supplement: Supplementary file 2 — Supplementary Table 1: Average binding free energies calculated from molecular dynamics (MD) simulations for moxidectin (MOX) and the chloride channel of Leishmania tropica. [file parasite-32-42-s2.pdf]

Supplementary Material Table 1: Average binding free energies calculated from the molecular dynamic (MD) simulation.

Complex	van der Waals (kJ/ mol)	Electrostatic (kJ/mol)	Polar energy (kJ/ mol)	Non-polar energy (kJ/mol)	Binding energy (kJ/mol)
MOX - CLC	-177.9	-13.7	78.2	-10.3	-123.7
