# Supplementary material for: Repositioning of moxidectin: a promising approach in cutaneous leishmaniasis therapy
Source: Parasite. 2025 Jul 4;32:42. doi: 10.1051/parasite/2025035 (PMC12232414; doi:10.1051/parasite/2025035)
Supplement: Supplementary file 3 — Supplementary Table 2: Top 10 upregulated and downregulated genes in L. tropica promastigotes following moxidectin (MOX) exposure, with corresponding log2 fold change values and statistical significance. [file parasite-32-42-s3.pdf]

Supplementary Material Table 2: Top 10 upregulated and downregulated genes in promastigotes following MOX exposure.

Gene ID	Gene name	Log2 fold change	p_value
XLOC_007420	ABC transporter family protein	5.07	0.005
XLOC_001204	ATP-binding cassette protein subfamily G, member 5, putative	4.89	5.00E-05
XLOC_005922	ATP-binding cassette protein subfamily B, member 2, putative	4.66	5.00E-05
XLOC_002721	ABC transporter-like protein	3.47	5.00E-05
XLOC_005712	HSP70 family protein	3.33	5.00E-05
XLOC_008835	Putative cytochrome P450 reductase	3.21	5.00E-05
XLOC_012021	Carbohydrate kinase, thermoresistant glucokinase family protein	3.2	0.0007
XLOC_007421	Conserved hypothetical protein	3.12	5.00E-05
XLOC_005236	Putative ATP-binding cassette protein subfamily A, member 6	3.04	5.00E-05
XLOC_004256	Putative ATP-binding cassette protein subfamily A, member 7	3.0	5.00E-05
XLOC_001801	Chloride channel protein, putative	-2.83	5.00E-05
XLOC_005336	Putative protein kinase	-2.83	0.0001
XLOC_005891	Kinesin, putative	-2.9	5.00E-05
XLOC_007542	N-Ethylmaleimide reductase-like protein	-2.94	0.0008
XLOC_006383	Conserved hypothetical protein	-2.99	5.00E-05
XLOC_011035	Putative calpain-like cysteine peptidase	-3.06	0.0007
XLOC_002844	Helicase-like protein	-3.1	5.00E-05
XLOC_012343	Hypothetical protein, unknown function	-3.29	0.0002
XLOC_001908	Putative calpain-like cysteine peptidase	-3.59	5.00E-05
XLOC_009739	Hypothetical protein, unknown function	-4.05	5.00E-05
