# Supplementary material for: Repositioning of moxidectin: a promising approach in cutaneous leishmaniasis therapy
Source: Parasite. 2025 Jul 4;32:42. doi: 10.1051/parasite/2025035 (PMC12232414; doi:10.1051/parasite/2025035)
Supplement: Supplementary file 4 — Supplementary Table 3: Top 10 upregulated and downregulated genes in L. tropica intracellular amastigotes following moxidectin (MOX) exposure, with corresponding log2 fold change values and statistical significance. [file parasite-32-42-s4.pdf]

Supplementary Material Table 3: Top 10 upregulated and downregulated genes in intracellular amastigotes following MOX exposure.

Gene ID	Gene name	Log2 fold-change	p_value
XLOC_007420	ABC transporter family protein	5.14	0.004
XLOC_001204	ATP-binding cassette protein subfamily G, member 5, putative	4.8	4.91E-05
XLOC_005922	ATP-binding cassette protein subfamily B, member 2, putative	4.64	5.32E-05
XLOC_002721	ABC transporter-like protein	3.51	4.71E-05
XLOC_005712	HSP70 family protein	3.36	5.26E-05
XLOC_008835	Putative cytochrome P450 reductase	3.26	5.19E-05
XLOC_012021	Carbohydrate kinase, thermoresistant glucokinase family protein	3.2	0.0007
XLOC_004256	Putative ATP-binding cassette protein subfamily A, member 7	3.02	4.85E-05
XLOC_002196	Putative ATP-binding cassette protein subfamily A, member 5	2.9	0.0004
XLOC_003422	Conserved hypothetical protein	2.6	5.00E-05
XLOC_002793	Conserved hypothetical protein	-2.87	0.001
XLOC_007194	Uncharacterized protein	-2.88	4.67E-05
XLOC_005336	Putative protein kinase	-2.9	9.38E-05
XLOC_007542	N-Ethylmaleimide reductase-like protein	-3.0	0.0008
XLOC_006383	Conserved hypothetical protein	-3.07	4.83E-05
XLOC_011035	Putative calpain-like cysteine peptidase	-3.11	0.0007
XLOC_002844	Helicase-like protein	-3.16	4.79E-05
XLOC_012343	Hypothetical protein, unknown function	-3.18	0.0002
XLOC_001908	Putative calpain-like cysteine peptidase	-3.63	4.92E-05
XLOC_009739	Hypothetical protein, unknown function	-3.98	5.08E-05
