# Supplementary material for: Repositioning of moxidectin: a promising approach in cutaneous leishmaniasis therapy
Source: Parasite. 2025 Jul 4;32:42. doi: 10.1051/parasite/2025035 (PMC12232414; doi:10.1051/parasite/2025035)
Supplement: Supplementary file 5 — Supplementary Table 4: List of differentially expressed genes within the seven assembled gene sets, including ABC transporters, HSP70, cytochrome c oxidase, NADH-cytochrome b5 reductase, aquaporins, major facilitator superfamily (MFS), and chloride channels. [file parasite-32-42-s5.pdf]

Supplementary Material Table 4: Gene IDs of the differentially expressed genes within the seven assembled gene sets.

Gene ID	Gene set
XLOC_000240	ABC transporters
XLOC_000241	
XLOC_001031	
XLOC_001204	
XLOC_001404	
XLOC_001831	
XLOC_001832	
XLOC_002196	
XLOC_002654	
XLOC_002721	
XLOC_003193	
XLOC_003194	
XLOC_003553	
XLOC_003693	
XLOC_003829	
XLOC_004331	
XLOC_005236	
XLOC_005323	
XLOC_005324	
XLOC_005325	
XLOC_005326	
XLOC_005327	
XLOC_005328	
XLOC_007289	
XLOC_007420	
XLOC_008048	
XLOC_008049	
XLOC_008050	
XLOC_008361	
XLOC_009921	
XLOC_010015	
XLOC_010054	
XLOC_010055	
XLOC_010101	
XLOC_010647	
XLOC_000147	HSP70
XLOC_001596	
XLOC_001962	
XLOC_002975	
XLOC_005712	
XLOC_005713	

XLOC_000428	**Cytochrome c oxidase**
XLOC_002176	
XLOC_002177	
XLOC_002178	
XLOC_002698	
XLOC_003504	
XLOC_006002	
XLOC_007380	
XLOC_007633	
XLOC_010686	
XLOC_004582	**Putative NADH-cytochrome b5 reductase**
XLOC_005072	
XLOC_005073	
XLOC_010127	
XLOC_011506	
XLOC_001024	**Aquaporins**
XLOC_003369	
XLOC_003498	
XLOC_003499	
XLOC_010007	
XLOC_000604	**Major Facilitator Superfamily protein**
XLOC_001168	
XLOC_001531	
XLOC_001559	
XLOC_001643	
XLOC_001864	
XLOC_002441	
XLOC_002918	
XLOC_003278	
XLOC_003419	
XLOC_003420	
XLOC_003421	
XLOC_003422	
XLOC_003901	
XLOC_004145	
XLOC_004259	
XLOC_007223	
XLOC_007527	
XLOC_007652	
XLOC_007840	
XLOC_008119	
XLOC_008167	
XLOC_008168	
XLOC_008681	
XLOC_008752	

XLOC_009770	
XLOC_009949	
XLOC_010476	
XLOC_010683	
XLOC_010684	
XLOC_001801	**Chloride channel proteins**
XLOC_004222	
XLOC_008946	
